# Adaptation to Long-Term Nitrogen Starvation in a Biocrust-Derived Microalga *Vischeria* sp. WL1: Insights into Cell Wall Features and Desiccation Resistance

**DOI:** 10.3390/microorganisms13040903

**Published:** 2025-04-14

**Authors:** Wensheng Liang, Xiang Gao, Yang She, Xin Jing, Xiaolong Yuan, Derui Zhu

**Affiliations:** 1 School of Food and Biological Engineering, Shaanxi University of Science & Technology, Xi’an 710021, China; 2 Research Center of Basic Medical Science, Medical College, Qinghai University, Xining 810016, China

**Keywords:** terrestrial microalgae, nitrogen deficiency, cell wall, desiccation resistance, environmental adaptation

## Abstract

In drylands, microalgae dwelling in the biocrust are inevitably confronted with nitrogen deficiency and desiccation stress, despite the protection afforded by the soil biological complex. However, the environmental adaptive features and mechanisms of these microalgae remain largely unknown. In this study, we explored the adaptive changes of a biocrust-derived unicellular microalga, *Vischeria* sp. WL1 (Eustigmatophyceae), in the face of long-term nitrogen deficiency. Attention was focused on the alterations in cell wall properties and the associated desiccation resistance. After exposure to long-term nitrogen deficiency, the cell walls of *Vischeria* sp. WL1 thickened substantially, accompanied by enhanced rigidity and an improvement in desiccation resistance. In contrast, *Vischeria* sp. WL1 cells cultivated under nitrogen-replete conditions were highly vulnerable to desiccation stress. Additional cell wall alterations after nitrogen starvation included distinct surface sculpturing, variations in monosaccharide composition, and changes in functional groups. Collectively, this study provides valuable insights into the survival strategies of biocrust-derived microalgae in nitrogen-deficient dryland environments.

## 1. Introduction

Nitrogen constitutes one of the principal nutrients indispensable for the growth of all living organisms. It plays a fundamental role in the biosynthesis of proteins, nucleic acids, and chlorophyll. Although nitrogen (N_2_) is highly abundant in the atmosphere, bioavailable forms are mainly combined nitrogen resources, such as ammonium and nitrate, which are scarce in nature [1]. Consequently, optimal nitrogen conditions for the growth of plants and algae are generally provided by human activities. Compared with the ideal growth state under nitrogen-replete conditions, a reduced nitrogen supply is commonly regarded as nitrogen deficiency (nitrogen starvation or nitrogen stress). In the wild, it is prevalent that organisms constantly experience nitrogen starvation. Hence, exploring long-term low-nitrogen or even nitrogen-free cultivation is crucial for understanding the survival strategies of organisms in the natural environment.

Microalgae are an important group of photosynthetic microorganisms inhabiting diverse ecosystems. They are characterized by high metabolic flexibility, excellent adaptability to environmental stresses, and great potential for generating biologically valuable products [2]. Technically, nitrogen deficiency is often adopted to induce the production of algae-based metabolites such as astaxanthin, β-carotene, and triacylglycerols [3,4,5]. The underlying mechanisms involve a shift in resource allocation from nitrogen-rich compounds to carbon-rich molecules [6]. Other adaptive responses manifest in morphological, physiological, biochemical, and ultrastructural alterations [7,8,9,10,11,12]. For example, nitrogen starvation typically results in the degradation of nitrogenous compounds (e.g., protein, amino acids, and chlorophyll), impairs photosynthesis and growth, and causes the thickening of the cell wall as well as the alteration in cell wall composition.

Although most microalgae thrive in aquatic environments, numerous species also grow under aeroterrestrial conditions. These terrestrial species must endure periodic dehydration and rehydration or long-term desiccation, which can at least trigger mechanical stress. Cell wall remodeling or thickening induced by nitrogen starvation is thought to be positively associated with desiccation resistance through (1) the formation of thick and rigid cell walls that safeguard the integrity of the cell or (2) the formation of extremely thin and flexible walls that permit water loss [13]. In some microalgae, the thickening of the cell wall can enhance its rigidity and mechanical resistance [8]. However, a flexible cell wall appears to be more conducive to preventing mechanical stress during dehydration. Biochemical remodeling of the cell wall can enhance its flexibility, allowing regulated shrinkage and expansion of cells. It has been reported that desiccation leads to progressive cell shrinkage and deformation in lichen-forming microalgae, yet this process is rapidly reversed once water becomes available [9]. In mosses, desiccation tolerance is independent of cell wall thickness but related to cell wall elasticity [14]. In addition, flexible cell walls have also been found in cells with a relatively rigid cell wall [9,13,15,16]. Thus, cell wall thickening helps to increase rigidity, yet a rigid cell wall does not necessarily restrict its flexibility. The association of cell wall dynamics and desiccation tolerance may be species-specific in plants and microalgae.

In terrestrial arid regions, typically characterized by nitrogen deficiency and aridity, microalgae have to develop various adaptive mechanisms, including dynamic changes in the cell wall [9,17]. Unlike free-living microalgae, some microalgae form a close connection with soil particles and become a part of biological soil crusts (biocrusts). In their native habitats, biocrust microalgae have to endure long-term nitrogen deficiency and desiccation stress, although the soil biological complex can provide them with a certain degree of protection. Previous studies on nitrogen starvation in microalgae, including those of Eustigmatophyceae, primarily involved short-term treatments (less than one month) or used low-concentration nitrogen resources [4,7,8,18]. Although long-term cultivation (more than 150 days) of *Nannochloropsis gaditana* has been reported, it was conducted in a semi-continuous mode with a reduced nitrogen concentration (2.65 mM NaNO_3_) [11]. In this regard, the adaptive changes or responses in the cell wall dynamics of biocrust-derived microalgae remain largely unclear.

Previously, we isolated a new strain of Eustigmatophyceae, *Vischeria* sp. WL1, from a dryland biocrust [19]. This strain has the typical features of Eustigmatophytes, such as the ability to accumulate oil rich in eicosapentaenoic acid [20]. Also, nitrogen deficiency can induce oil accumulation in this strain. We observed that when co-cultivated with the nitrogen-fixing cyanobacterium *Leptolyngbya* sp. WL1 (NCBI no. OL701273.1) in nitrogen-free medium, single cells of *Vischeria* sp. WL1 aggregated and were enclosed by the filaments of *Leptolyngbya* sp. WL1; once nitrogen resource was added to the medium, the former quickly dispersed. Additionally, we found that this strain can endure extremely long-term nitrogen starvation during shake cultivation (e.g., 3–6 months). Hence, it is likely to be an evolutionarily distinctive strain within the Eustigmatophyceae. In this study, we aimed to investigate the cell wall features and the associated desiccation resistance in *Vischeria* sp. WL1 under long-term nitrogen starvation. This research would provide valuable insights into the nitrogen-starvation adaptation properties and potential mechanisms of this strain.

## 2. Materials and Methods

### 2.1. The Algal Strain and Nitrogen Starvation Treatment

The terrestrial microalga *Vischeria* sp. WL1 was isolated from a biological soil crust in northwest China [19]. It was regularly cultivated in BG11 (nitrogen-replete) liquid medium under continuous LED white light with an intensity of 60 μmol photons m^−2^ s ^−1^ at 25 °C in a shaker (130 rpm). Logarithmic-phase *Vischeria* sp. WL1 cells proliferated in BG11 medium were collected by centrifugation (1776× *g*, 5 min). For the nitrogen starvation treatment, the resulting pellets were rinsed twice with BG11_0_ (nitrogen-deplete) medium. Then, the rinsed cells were transferred to 150 mL of BG11_0_ medium (with a final OD_750_ of approximately 0.4) for 60 days of cultivation. At the end of the first 60-day cultivation, they were similarly collected and subjected to another two periods of 60-day cultivation, amounting to a total of 180 days of nitrogen starvation. BG11-cultivated cells and BG11_0_-cultivated cells (hereinafter referred to as nitrogen-replete cells and nitrogen-starved cells, respectively) were collected and subjected to various comparative studies.

### 2.2. Microscopic, SEM, and TEM Observations of Cells

For microscopic observation, freshly collected cells were placed onto glass slides and allowed to dry naturally at room temperature (25 °C). At 0, 6, 24, and 48 h, the cells on each slide were rehydrated using a drop of sterile water to prepare temporary slides for examination under an optical microscope.

For scanning electron microscopy (SEM) observation, the collected cells were stored at −20 °C for several hours and subsequently vacuum freeze-dried. The dehydrated samples were sputter-coated with a gold layer and then observed with a FEI Q45 + EDAX Octane Prime electron microscope (FEI and EDAX, Pleasanton, CA, USA).

For transmission electron microscopy (TEM) observation, the collected cells were fixed with 2.5% glutaraldehyde solution (pH 7.4) and then post-fixed in 1% OsO_4_. Subsequently, the samples were dehydrated with a graded ethanol series and embedded in Epon812 (Oken shoji, Tokyo, Japan). Ultrathin sections (70 nm in thickness) were prepared and examined with a Hitachi-HT7800 electron microscope (Hitachi, Tokyo, Japan).

### 2.3. Cell Wall Extraction

Cell cultures were centrifuged at 895× *g* for 5 min to separate the floating cell wall residues generated from dead or mother cells during reproduction [18]. The pelleted cells were resuspended in deionized water, transferred to a 2 mL centrifuge tube, and disrupted with steel balls (6 mm in diameter) using a Wonbio-R (China) high-throughput tissue grinder (60 Hz, 40 times, 30 s operation each time). After disruption, the cell wall fragments were collected by centrifugation (6180× *g*, 10 min) and rinsed with 5 mL of chloroform and 5 mL of deionized water. The upper aqueous phases containing cell wall fragments were collected and subjected to another round of centrifugation. The resulting white cell wall pellets were rinsed three times with absolute ethanol and then twice with deionized water, followed by freeze-drying to obtain dry materials.

### 2.4. SEM and AFM Observations of the Extracted Cell Wall

The SEM observation was conducted as described above. Apart from SEM observation, the surface topography and roughness of cell wall materials were also examined by atomic force microscopy (AFM) [21]. In brief, the cell wall materials were suspended in water and then dropped onto a mica sheet for air-drying. The dried samples were observed using an Agilent 5100 microscope (Agilent, Santa Clara, CA, USA).

### 2.5. XRD and FTIR Analyses of the Extracted Cell Wall

X-ray diffraction (XRD) is a nondestructive technique for assessing the crystallinity of solid materials like cellulose [22,23]. The cell wall materials were compressed into tablets for analysis using a Rigaku SmartLab 9kW XRD spectrometer (Rigaku, Tokyo, Japan).

Fourier transform infrared spectroscopy (FTIR) is employed to identify the functional groups within the materials [24]. The cell wall materials were ground together with dry KBr powder and then compressed into tablets for analysis by a Bruker VECTOR-22 spectrometer (Bruker, Ettlingen, Germany). Spectra were recorded over the range of 4000–400 cm^−1^ at a spectral resolution of 4 cm^−1^.

### 2.6. Analysis of the Monosaccharide Composition of Cell Wall

The monosaccharide composition of cell walls was determined with a ThermoFisher ICS5000 ion chromatography (Thermo Fisher Scientific, Waltham, MA, USA) equipped with a Dionex Carbopac PA10 column, as previously described [25]. Briefly, cell wall materials were hydrolyzed with 3 M trifluoroacetic acid at 121 °C for 3 h. The monosaccharide standards were glucose, galactose, mannose, fucose, rhamnose, arabinose, xylose, fructose, ribose, galacturonic acid, and glucuronic acid. The relative monosaccharide compositions (in mol%) of the cell walls were calculated.

### 2.7. Cell Survival Observation Through Fluorescence Dye Staining

The nitrogen-replete or nitrogen-starved cells were collected by centrifugation and then naturally dried at room temperature (25 °C) for 0, 0.5, and 1 h, respectively. Subsequently, the cells were transferred into 1 mL of deionized water and stained with 1.5 μL of SYTO 9 fluorescent dye (3.34 mM stock solution in DMSO) and 1.5 μL of Propidium Iodide (20 mM) [26]. After staining for 15 min, the cells were collected by centrifugation and rinsed to eliminate the floating color. Then, the cells were observed using an Olympus FV10i Laser Scanning Confocal Microscopy (Olympus, Tokyo, Japan). The excitation peaks for SYTO 9-DNA and Propidium Iodide-DNA are 485 nm and 535 nm, respectively, while the emission peaks are 498 nm and 615 nm, respectively. Living cells exhibited green fluorescence, whereas dead cells showed red fluorescence.

### 2.8. Cell Survival Testing on Solid Medium

Aliquots of 10 μL nitrogen-replete or nitrogen-starved cell cultures were collected and dropped onto sterilized nitrocellulose membranes, followed by natural air-drying for 0, 0.5, 1, and 4 h, respectively. Then, the membranes were transferred to BG11 agar plates. Photographs of the cell colonies (whether proliferating or dead) on the membranes were taken on days 0, 20, 27, and 41.

## 3. Results

### 3.1. Morphological Integrity of Cells After the Drying Treatment

**Figure 1:**
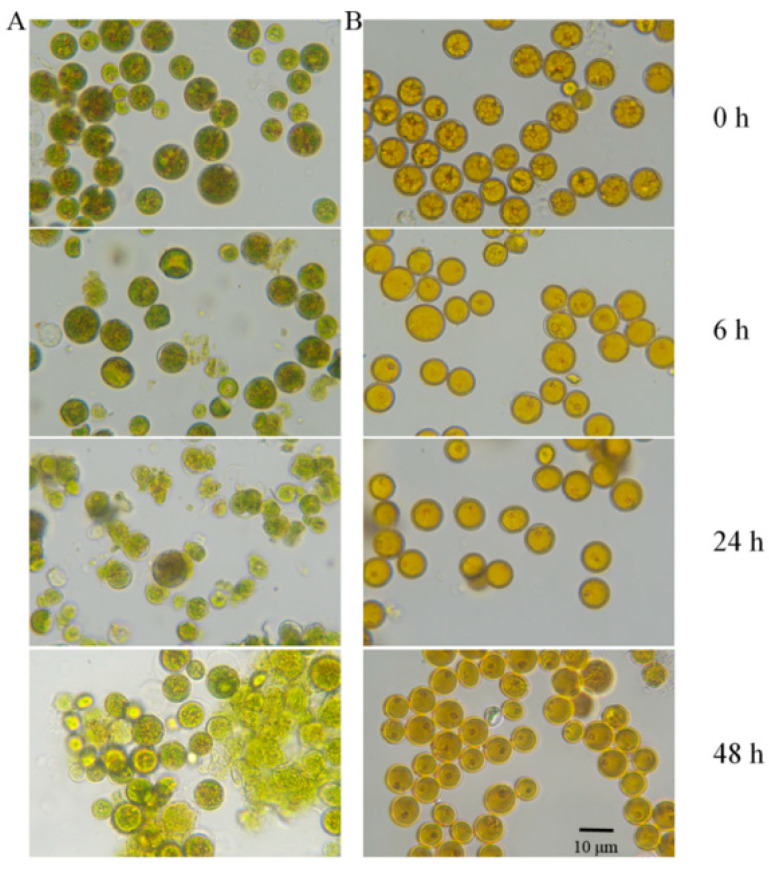
Microscopic observation of *Vischeria* sp. WL1 cells after air-drying and subsequent rehydration. (**A**) Nitrogen-replete cells; (**B**) nitrogen-starved cells. The cells were observed on temporary glass slides under an optical microscope. Scale bar, 10 μm.

After being cultivated for 180 days in BG11_0_ medium, the nitrogen-starved *Vischeria* sp. WL1 cells in the glass flask exhibited a yellow color, which was in contrast to the nitrogen-replete cells, which displayed a green color. The nitrogen-starved cells became much closer to a resting state. However, their photophysiological activity, in terms of the maximum photochemical efficiency of photosystem II (Fv/Fm), was detected to be still at a high level (above 0.65). The logarithmic-phase cells cultured in BG11 medium (nitrogen-replete cells) were collected for comparison. Both types of cells were subjected to air-drying for different times (within 48 h) and then rehydrated for microscopic observation (Figure 1). For the nitrogen-replete cells, cell cracks appeared after the treatments. Moreover, as the drying time was prolonged, the number of cracked cells increased significantly. In contrast, throughout all the drying periods, no cracks were observed in the nitrogen-starved cells. These results suggest that long-term nitrogen starvation markedly reinforces the cell wall rigidity of *Vischeria* sp. WL1.

### 3.2. Cell Observation by SEM and TEM

**Figure 2:**
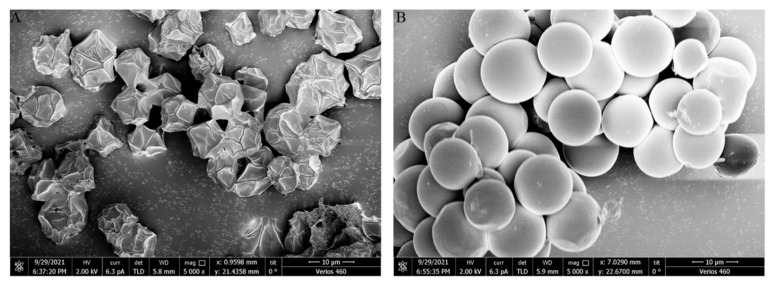
SEM observation of the freeze-dried *Vischeria* sp. WL1 cells. (**A**) Nitrogen-replete cells; (**B**) nitrogen-starved cells.

Freeze-drying can impose stresses from both freezing and water loss. After freeze-drying, the cell surface and morphology of nitrogen-replete and nitrogen-starved *Vischeria* sp. WL1 cells were imaged using SEM (Figure 2). The nitrogen-replete cells shrank with polygonal wrinkles, while the nitrogen-starved cells retained their spherical shape and smooth surface. This observation further suggests that the nitrogen-starved cells possess a more rigid cell wall than the nitrogen-replete cells.

**Figure 3:**
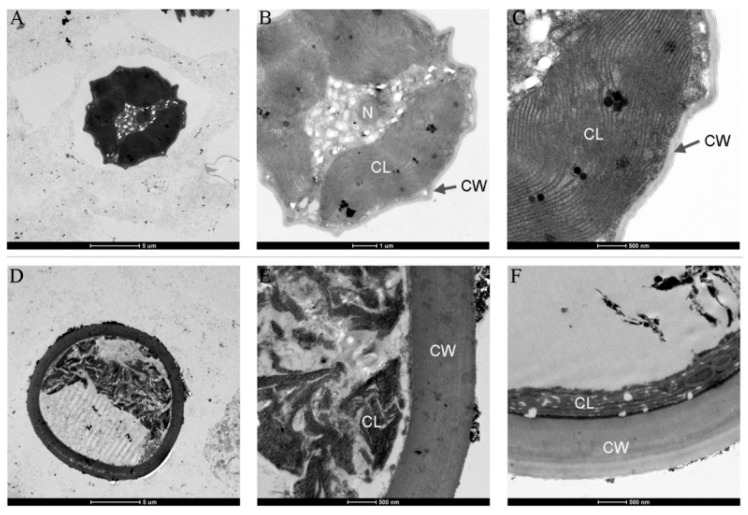
TEM observation of freeze-dried *Vischeria* sp. WL1 cells. (**A**–**C**) Nitrogen-replete cells; (**D**–**F**) nitrogen-starved cells. N: Nucleus. CL: Chloroplast. CW: Cell Wall.

The cellular ultrastructure was further compared between nitrogen-replete and nitrogen-starved cells (Figure 3). It was observed that the nitrogen-replete cells contained a large number of photosynthetic lamellae, while the photosynthetic lamellae in the nitrogen-starved cells were disordered and fractured. Additionally, the cell wall of the nitrogen-starved cells was thicker than 1 μm, which is approximately seven times that of the nitrogen-replete cells (150 nm). It was reported that the cell wall thicknesses of three microalgal strains (*Nannochloropsis* sp., *Chlorococcum* sp., and *Chlorococcum* sp.) were 12 nm, 52 nm, and 387 nm, respectively, and nitrogen depletion only led to a cell wall thickening of no more than 70% [8]. The notably thickened cell wall might have contributed to endowing the nitrogen-starved *Vischeria* sp. WL1 cells with high rigidity.

### 3.3. SEM and AFM Observations of Cell Walls

**Figure 4:**
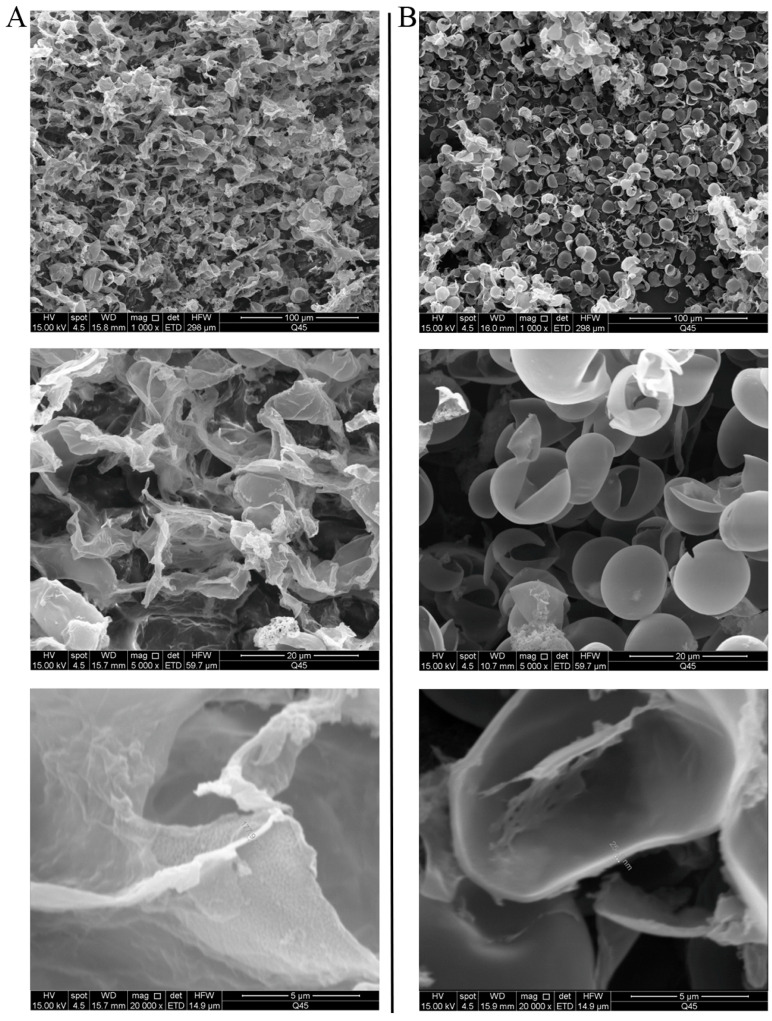
SEM observation of the extracted cell walls. (**A**) Cell walls of nitrogen-replete cells; (**B**) cell walls of nitrogen-starved cells.

**Figure 5:**
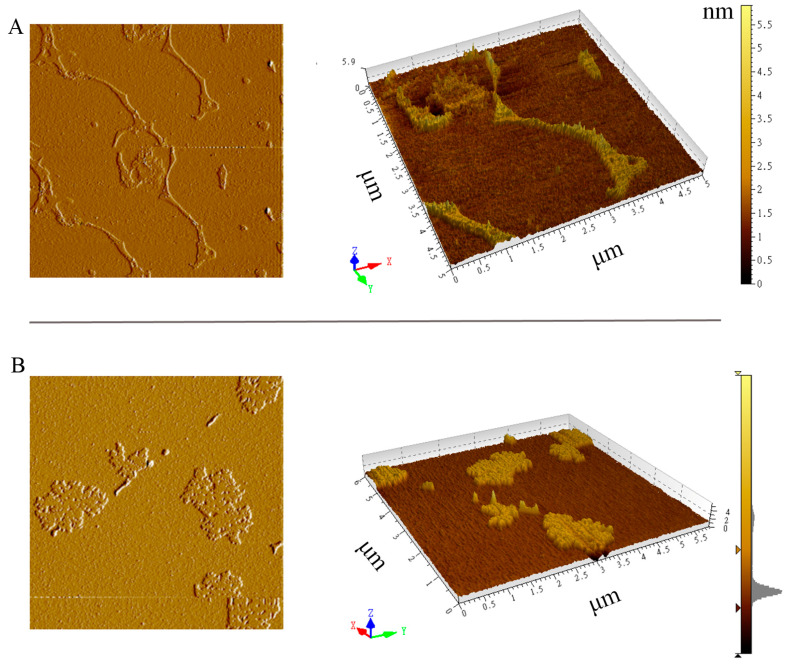
AFM observation of the cell wall sculptures. (**A**) Cell walls of nitrogen-replete cells; (**B**) cell walls of nitrogen-starved cells. Left panels, two-dimensional topographic maps; right panels, three-dimensional topographic maps. The bars indicate the height (nm) of the sculptures on the extracted cell walls.

Cell walls were extracted from nitrogen-replete and nitrogen-starved cells for fine-scale observations using SEM and AFM (Figure 4 and Figure 5). During the extraction process, cells were disrupted by steel balls through vibration. As shown in Figure 4, the cell walls of the nitrogen-replete cells were clearly fragmented and wrinkled, while those of the nitrogen-starved cells mostly resembled open shells. This cell wall fissure might facilitate daughter cell release during vegetative propagation. In addition, consistent with Figure 3, cell walls from the nitrogen-starved cells were much thicker. In Eustigmatophyceae, some members of the Goniochloridales have ornamentation with sculpting (e.g., warts, bulges, or papillae) on their cell walls [20]. We thus also examined the surface topography of cell walls in both types of cells (Figure 5). Ridge-like protrusions were distributed on the cell walls of the nitrogen-replete cells, while on the cell walls of the nitrogen-starved cells, the protrusions were flat and mound-like. Thus, at a finer level of observation, the cell wall of *Vischeria* sp. WL1 is not smooth and featureless.

### 3.4. XRD and FTIR Analyses of Cell Walls

**Figure 6:**
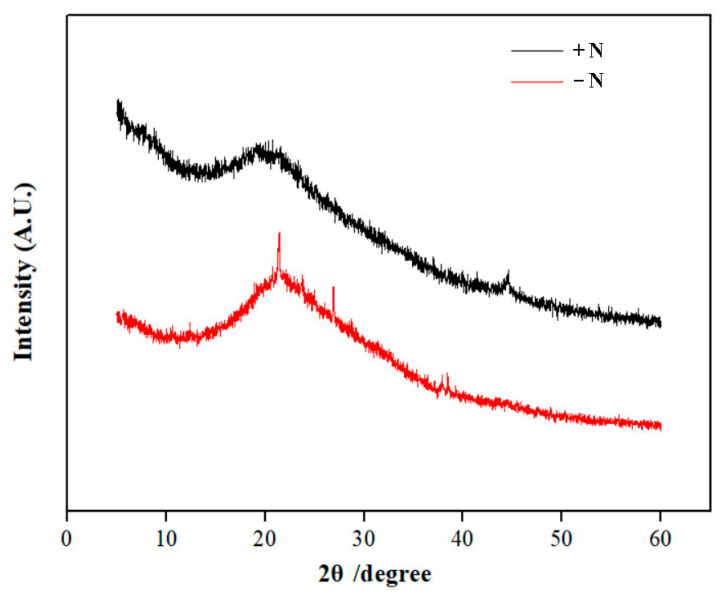
XRD analysis of the extracted cell walls. +N, cell walls of nitrogen-replete cells; -N, cell walls of nitrogen-starved cells.

XRD analysis was employed to compare the crystallinity of cell walls between nitrogen-replete and nitrogen-starved cells (Figure 6). Evident differences were observed in both the 2θ position and the shape of the diffraction peaks. The cell walls of nitrogen-starved cells had typical peaks (2θ) at 21.52° and 28.38°, while both peaks were lacking in the cell walls of nitrogen-replete cells. Thus, long-term nitrogen starvation resulted in a higher crystallinity of cell wall components.

**Figure 7:**
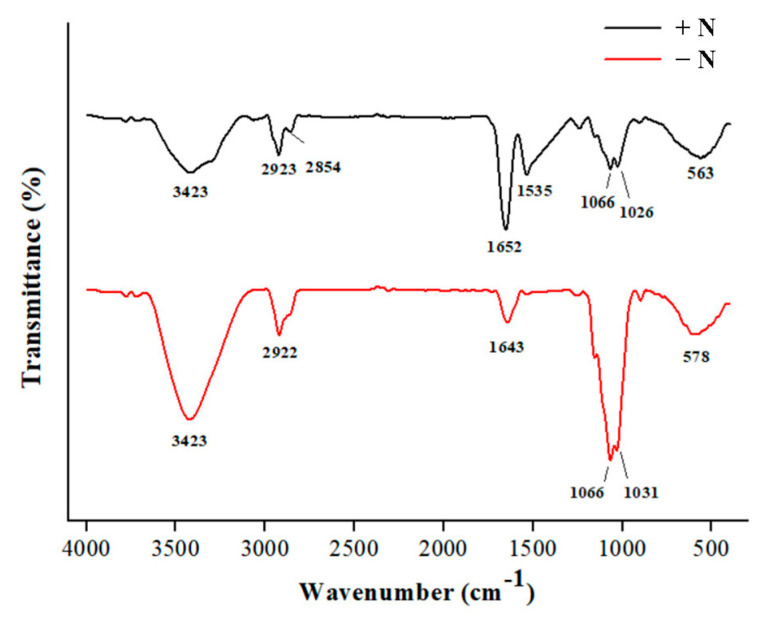
FTIR analysis of the extracted cell walls. +N, cell walls of nitrogen-replete cells; -N, cell walls of nitrogen-starved cells.

According to the FTIR spectra (Figure 7), three typical regions could be distinguished: region I (3500–2800 cm^−1^), region II (1700–1500 cm^−1^), and region III (1100–1000 cm^−1^). Region I (the X–H stretching region) is an indicator of cellulose [27,28]. The absorbance bands at 2923/2922 cm^–1^ and 2854 cm^–1^ are related to the C–H stretching. However, the 2854 cm^–1^ band disappeared in the cell wall of nitrogen-starved cells. The absorbance band at 1643 cm^–1^ or 1652 cm^–1^ in region II (the H–O–H bending vibration) may indicate the presence of microcrystalline cellulose [29]. Region III (the C–O stretching and C–C stretching) may suggest the presence of lignin or mannose-containing hemicellulose [28,29]. The potential protein compound, with the N–H bending of the amide II at 1535 cm^−1^ [29], vanished in the cell wall of nitrogen-starved cells. In addition, the relative heights of the absorbance bands at 3423 cm^–1^, 1652/1643 cm^–1^, and 1066 cm^−1^ differed obviously between the cell walls of nitrogen-replete and nitrogen-starved cells, demonstrating the different contents of cell wall components.

### 3.5. The Monosaccharide Composition of Cell Walls

The monosaccharide compositions of cell walls from two types of cells were further analyzed (Table 1). Interestingly, only four kinds of monosaccharides were detected in their cell wall materials, with glucose being dominant. This composition is obviously different from those of some other Eustigmatophytes, such as *Trachydiscus guangdongensis* and *Nannochloropsis gaditana* [18,30]. The cell wall of *T. guangdongensis* consists of seven kinds of monosaccharides, with galactose being the predominant one [18]. In the cell wall of *N. gaditana*, seven kinds of monosaccharides were also detected, and the glucose content is approximately 98% [30]. After nitrogen starvation, the relative proportions of the four monosaccharides in *Vischeria* sp. WL1 were altered, with only the proportion of glucose showing a relative increase.

### 3.6. Comparison of Desiccation Resistance

**Figure 8:**
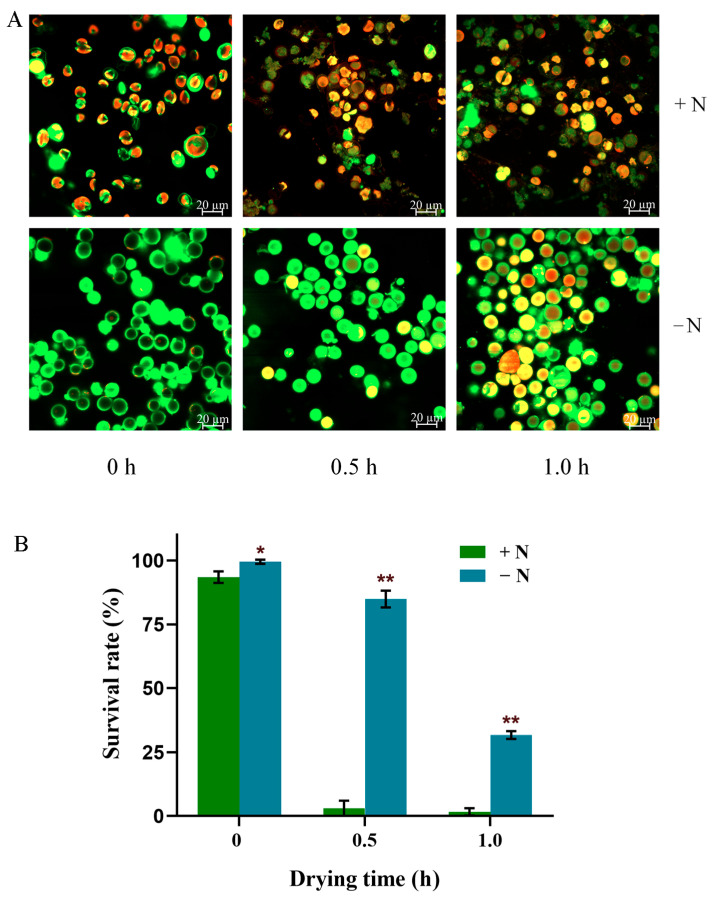
Fluorescence staining (**A**) and cell survival rate (**B**) of nitrogen-replete and nitrogen-starved cells after natural drying for 0, 0.5, and 1.0 h, respectively. Data shown are the mean ± SD (n = 3). * significant difference (*p* < 0.05, *t*-test), ** highly significant difference (*p* < 0.01, *t*-test).

**Figure 9:**
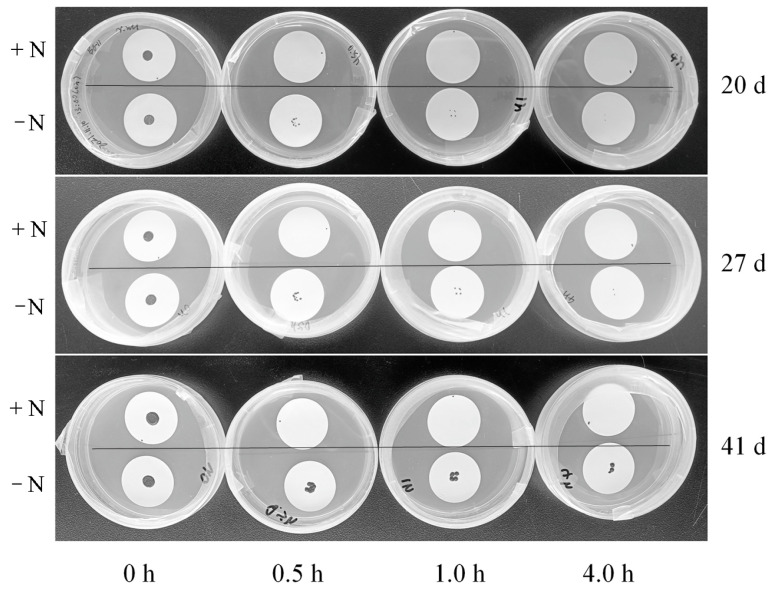
The survival of nitrogen-replete and nitrogen-starved cells on BG11 agar plates. Prior to cultivation, the cells were exposed to natural air-drying for 0, 0.5, 1.0, and 4.0 h, respectively.

Cell wall thickness is associated to a certain extent with the mechanical resistance [8]. Dehydration is one of the ways that can cause mechanical stress on cells. Thus, we further compared the desiccation resistance of nitrogen-replete and nitrogen-starved cells (Figure 8 and Figure 9). Upon short-term drying treatments (within 1 h), the nitrogen-replete cells appeared to be highly sensitive to desiccation, whereas the nitrogen-starved cells were more resistant (Figure 8). Following the 1 h treatment, nearly all nitrogen-replete cells died, while more than 30% of the nitrogen-starved cells remained alive. Furthermore, we conducted drying treatments (within 4 h) followed by cultivation (Figure 9). The results indicated that the nitrogen-starved cells could survive up to 4 h of desiccation, while the nitrogen-replete cells could not survive 0.5 h of desiccation. Nevertheless, the nitrogen-starved cells did not exhibit high resistance to desiccation stress, as shown by the 1 h treatment in Figure 8 and the 4 h treatment in Figure 9. Based on these observations, the thickening of the cell wall merely served as one of the factors contributing to the desiccation resistance of *Vischeria* sp. WL1 cells.

## 4. Discussion

In drylands, biocrusts are formed by the adhesion of soil particles to the exopolysaccharides secreted by cyanobacterial and green algal communities [31]. *Vischeria* sp. WL1 was previously isolated from a dryland biocrust in our laboratory [19]. After long-term nitrogen starvation, its resistance to desiccation was improved but was still very limited (Figure 8 and Figure 9). Therefore, it is impossible for this strain to survive in a free-living manner in the dryland environment. In its native habitat, the soil is extremely nitrogen-deficient, with less than 0.04% total nitrogen [32]. Usually, when cultivated under long-term nitrogen deficiency, most microalgae will either die or enter a dormant state (for instance, the diatom *Chaetoceros socialis* forms resting spores) [33]. In this study, *Vischeria* sp. WL1 survived the 180-day nitrogen-deficiency cultivation. After 180 days, its photophysiological activity could still be detected, with an Fv/Fm value above 0.65; also, as shown in Figure 8 and Figure 9, the nitrogen-starved cells exhibited survival capacity. It is worth noting that the nitrogen content in cells after two months of nitrogen starvation had already dropped to a level close to that after six months. These imply that long-term nitrogen starvation (up to 180 days) might result in a metabolically weak state rather than a resting or dead state. This physiologically active state would help the cells quickly restore full vitality when encountering external nitrogen sources. Previously, the morphological, physiological, and ultrastructural features of such strains derived from biocrusts have rarely been studied. By focusing on cell wall characteristics and desiccation resistance, this study has offered valuable insights into the survival strategies of biocrust-derived microalgae in the dryland environment.

Apart from oil accumulation, cell wall thickening constitutes another significant feature in microalgae under nitrogen depletion [8]. In line with this observation, the cell wall of *Vischeria* sp. WL1 was substantially thickened (more than 1 μm), much thicker than those of other microalgae such as *Nannochloropsis* sp. and *Haematococcus pluvialis* [8,34,35]. In *Nannochloropsis*, the cell wall thickness varied from 63 to 119 nm, depending on the species [34]; in *Haematococcus pluvialis*, a relatively thicker cell wall (up to 0.8 μm) was observed [35]. After nitrogen starvation, some biochemical features, as reflected by XRD and FTIR analyses, were also altered in *Vischeria* sp. WL1 (Figure 5 and Figure 6). Other fascinating findings involve the monosaccharide composition of the cell wall of *Vischeria* sp. WL1, where only four monosaccharides were detected (Table 1). Glucose is the principal component of the cell wall, suggesting that the cell wall of *Vischeria* sp. WL1 might be mainly composed of cellulose, similar to *T. guangdongensis* [18]. The rigidity of the cell wall was greatly enhanced after long-term nitrogen starvation, as witnessed in the morphological and ultrastructural dynamics (Figure 1, Figure 2 and Figure 4). The increased cell wall thickness may have contributed to the improved rigidity. Although *Vischeria* sp. WL1 can be safeguarded by the soil biological complex, it is still inevitably subjected to periodic desiccation. Thus, a thicker and more rigid cell wall is evidently advantageous for coping with this harsh situation.

Depending on the species or strain, either a thicker or a more flexible (perhaps not thick) cell wall can facilitate a cell’s resistance to mechanical stress resulting from dehydration [8,9,14]. However, mechanical resistance is not the whole mechanism for coping with desiccation stress. Physiological and molecular adaptations are also of crucial importance [9,13]. Thick cell walls and intense rigidity were observed in the nitrogen-starved *Vischeria* sp. WL1 cells (Figure 1, Figure 2, Figure 3 and Figure 4). In addition, the change from ridge-like sculptures to flat mound-like ones on the cell wall upon nitrogen deficiency (Figure 5) might also contribute to the reinforcement of cell wall rigidity. This mechanical strengthening of the cell wall was apparent for retaining the morphological integrity of cells (Figure 1 and Figure 2). The cell wall of nitrogen-replete cells was more flexible, yet it did not assist the cells in resisting mechanical and desiccation stresses (Figure 1, Figure 2, Figure 8 and Figure 9). Although the cell wall of nitrogen-starved cells was thicker and more rigid, the cells only exhibited limited tolerance to desiccation stress (Figure 8 and Figure 9). Thus, a cell wall that is both flexible and rigid appears to be far more crucial for mechanical or desiccation resistance. In the previous exploratory test, we also observed that after freeze treatment at −20 °C, both nitrogen-replete and nitrogen-starved cells could not survive in BG11 medium. Therefore, in *Vischeria* sp. WL1, the thickening of the cell wall should mainly be an adaptive response to nitrogen deficiency and offer only a limited contribution to environmental stress resistance.

It has been reported that an increased frequency of summer rainfall can lead to rapid moss mortality in drylands [36]. Moreover, nitrogen deposition or addition can impact soil microbial growth, composition, and function, and these effects are often negative [37,38]. Our results on *Vischeria* sp. WL1 further show that a nitrogen-rich condition is not conducive to its environmental adaptation. Instead, persistent nitrogen starvation, regarded as a “normal” growth circumstance, has certain advantages in enhancing its resistance to desiccation stress. In the next steps, other physiological, biochemical, or molecular alterations taking place in *Vischeria* sp. WL1 upon extremely long-term nitrogen stress, as well as different nitrogen sources, remain to be explored.

## Figures and Tables

**Table 1:** Relative monosaccharide compositions of the cell walls in nitrogen-replete and nitrogen-starved cells.

Monosaccharides (mol %)	Glucose	Fucose	Mannose	Galactose
Nitrogen-replete cells	0.702	0.153	0.088	0.057
Nitrogen-starved cells	0.827	0.095	0.057	0.021

## Data Availability

All data supporting the findings of this study are included in the main article.
